# Performance of AI Tools in Citing Retracted Literature : Content Analysis

**DOI:** 10.2196/88766

**Published:** 2026-05-01

**Authors:** Sebastian Labenbacher, Maximilian Niederer, Sascha Hammer, Matthias Bader, Nikolaus Schreiber, Helmar Bornemann-Cimenti

**Affiliations:** 1 Department of Anesthesiology and Intensive Care Medicine, Medical University of Graz, Auenbruggerplatz 5, Graz, 8036, Austria, 43 316-385-81843

**Keywords:** artificial intelligence, AI, retraction of publication, retractions, scientific misconduct, evidence-based Practice, data accuracy, ethics

## Abstract

**Background:**

Generative artificial intelligence (GenAI) tools are increasingly used in scientific research to support literature searches, evidence synthesis, and manuscript preparation. While these systems promise substantial efficiency gains, concerns have emerged regarding their reliability, particularly their tendency to cite inaccurate, fabricated, or retracted literature. The unrecognized inclusion of retracted studies poses a serious risk to research integrity and evidence-based decision-making. Whether commonly used GenAI tools can reliably detect, exclude, or transparently communicate the retraction status of scientific publications remains unclear.

**Objective:**

This study aimed to evaluate the ability of freely available GenAI tools to correctly handle retracted scientific articles during literature searches. Primary and secondary outcomes focused on accuracy, reliability, and consistency in recognizing retracted literature.

**Methods:**

In this pragmatic trial, nine widely used free-access GenAI tools (ChatGPT 4, ChatGPT 5, Claude, Gemini, Perplexity, Microsoft Copilot, SciSpace, ScienceOS, and Consensus) were evaluated. Each tool was asked five predefined, standardized questions addressing topic overview, article identification, article summarization, and explicit assessment of retraction status. Overall, 15 retracted articles (the 10 most cited and 5 most recently retracted as of May 23, 2025) were selected from the Retraction Watch database. All questions were repeated twice to assess intratool consistency. Responses were independently rated as correct or incorrect by 2 researchers. Descriptive statistics summarized performance, and comparisons between general-purpose and research-focused AI tools were conducted using descriptive statistics. Interreviewer agreement was assessed using Cohen kappa coefficient.

**Results:**

None of the evaluated AI tools consistently handled retracted articles correctly. No model achieved perfect accuracy across all question sets. ChatGPT 5 performed best, defined by the primary outcome of achieving fully correct responses to all five predefined tasks (5/5) for the highest number of retracted articles, correctly answering all five questions for 8 of 15 articles (53.3%). Research-focused tools (SciSpace, ScienceOS, and Consensus) failed to produce a single fully correct response set. Retracted articles were frequently included in topic overviews without warning, with error rates exceeding 40% in several tools. When specifically asked about retraction status, most systems failed to provide correct or complete information. OpenEvidence only reported data for a subset of our retracted articles as it is only used in health care literature. It demonstrated strong performance in topic overviews but low accuracy in identifying retracted articles.

**Conclusions:**

Freely available GenAI tools are currently not able to detect, exclude, or appropriately flag retracted scientific literature. The widespread and confident reproduction of retracted studies represents a substantial threat to research integrity, particularly in medical and evidence-based fields. Until retraction-aware verification mechanisms are systematically integrated, independent source checking remains essential when using AI-assisted literature tools.

## Introduction

Artificial intelligence (AI) has gained substantial importance in scientific research in recent years, with growing use in text generation and automated knowledge retrieval [1,2]. Especially in health sciences, AI-based applications are being integrated into research workflows, and early evaluations suggest that these technologies can accelerate literature reviews, facilitate the synthesis of large datasets, and assist researchers and students in scientific communication [3,4]. An example of a generative artificial intelligence (GenAI) system developed for medical information gathering is OpenEvidence, which can exclusively be used by health care professionals. In particular, the ability of AI tools to efficiently process large volumes of information has been highlighted as a promising complement to established methods of evidence appraisal and synthesis [5,6].

At the same time, important limitations and risks of these technologies have become increasingly evident [7,8]. AI-assisted text generation may produce erroneous or fabricated references, thereby raising concerns about the integrity of GenAI-supported scientific work [9]. Beyond the creation of non-existent citations, the inadvertent inclusion of retracted publications represents a critical issue that can seriously undermine the reliability of evidence syntheses [10,11]. Recent reports have demonstrated that large language models (LLMs) frequently cite retracted or unreliable studies without acknowledgment, often presenting them with high linguistic confidence and apparent authority [12]. This behavior poses a direct threat to research integrity and may ultimately affect patient safety, particularly in evidence-based disciplines such as medicine, where retracted data can distort clinical conclusions.

Despite the rapid proliferation of AI tools for academic use, it remains unclear whether these systems can reliably detect, flag, or communicate the retraction status of scientific articles. Earlier investigations have focused primarily on single models or subject areas, leaving the broader cross-platform performance of GenAI largely unexplored [13].

Even AI tools marketed for scientific and health care research—such as those designed to assist literature searches or summarize findings—have not been systematically tested for their ability to recognize retracted work. Against this background, this study evaluated nine widely used GenAI systems, including ChatGPT 5, ChatGPT 4, Claude, Gemini, Perplexity, Microsoft Copilot, SciSpace, ScienceOS, and Consensus, with OpenEvidence analyzed separately as a medical literature–specific model. Using a predefined set of retracted clinical trials, each tool was assessed for factual accuracy, reliability, and risk of propagating retracted studies. The aim was to critically assess the reliability of AI-assisted literature searches and, based on these findings, to provide recommendations for their responsible and safe application in scientific practice.

## Methods

### Study Design

This study was designed as a pragmatic evaluation using freely accessible artificial intelligence tools (AI) for literature screening and summarization of articles with five predefined questions to assess how retracted articles are handled. The trial was registered during the data acquisition phase in the Open Science Framework (osf.io) [14].

The predefined questions were designed to outline a pathway for investigating a new topic of interest: providing an overview based on the article’s keywords, identifying the most important research articles related to the defined topic, and summarizing the retracted article. The final two questions were directly aimed at the retraction status of the respective article. Each response was scored as correct (1) or incorrect (0).

We used AI tools specifically designed for scientific work (Consensus Version July 2025, SciSpace Version 1.5.1, ScienceOS Version July 2025, OpenEvidence Version July 2025) as well as generic AI tools (ChatGPT Version 4 and 5, Microsoft Copilot Version 3.0, Gemini Version 2.5, Perplexity AI Version July 2025, Claude AI Version 3.5). AI tools with trial versions insufficient to perform all necessary research questions were excluded.

### Ethical Considerations

This study did not involve human participants, patient data, or identifiable personal information and was limited to the analysis of publicly available literature and AI-generated outputs. In accordance with institutional and international research ethics guidelines, ethics committee approval was not required.

### Questions

The following questions were used to guide the literature assessment:

1. Give me an overview on publications of “keywords of the article”
2. Give relevant articles regarding the topic of “keywords of the article”
3. Summarise the article “TITLE” published by “AUTHORS” for me
4. Is the article “TITLE” published by “AUTHORS” retracted?
5. Why is “TITLE” published by “AUTHORS” retracted?

The exact titles (excluding RETRACTED or any other details on the retraction status), authors, and keywords we used can be viewed in the supplement (Table S1 in Multimedia Appendix 1, [15-29]).

### Scoring and Rating of Responses

A binary scoring system (one point per question) was used to emphasize functional correctness in AI-assisted literature workflows, acknowledging that this approach sacrifices linguistic nuance. Responses using equivocal or nonspecific wording (eg, “controversial” or “heavily criticized” instead of explicitly stating retraction) were rated as incorrect, as such phrasing does not clearly state retraction status. This conservative approach was chosen to avoid overestimating AI performance where precise identification of retracted literature is critical.

### Retracted Articles

#### Overview

To ensure objectivity, we used the ten most cited, retracted articles found in the Retraction Watch website [30]. Furthermore, we used 5 articles, retracted closest to May 23, 2025 (extracted from the retraction-watch database [31]) to compare whether the number of citations or the date of retraction has an impact on our results [15-29]. Details of the included articles can be found in Table S1 in Multimedia Appendix 1 [15-29].

In the process of choosing the papers for our search, we did not find keywords for every article. Keywords were essential to our methods, and we therefore developed our own keywords for the articles. To ensure a uniform keyword generation process, we decided to let ChatGPT 4 choose the respective keywords for the articles. As ChatGPT is also a tool in evaluation, we performed an exploratory analysis of keyword generation restricted to general-purpose GenAI tools (ChatGPT 4, Claude, Gemini, and Perplexity), as these models were used for keyword generation in our study design. Research-focused AI tools were not included in this analysis.

#### Study Aims

Our primary aim was to assess how often AI tools correctly handled retracted articles. Correct handling was defined as providing accurate responses to all 5 predefined questions, including appropriate identification and summarization of the article and correct recognition and explanation of its retraction status.

#### Secondary Aim

The secondary aims of this study were as follows:

1. How often were retracted articles included in a topic overview (topic overviews include questions 1 and 2)
2. How often were the retracted articles included in the search pathway (search pathway includes questions 1, 2, and 3)
3. To analyze how often results differ when the same question is asked twice
4. To compare general AI tools (like ChatGPT) against AI tools specifically designed for research (like ScienceOS or Consensus)

#### Exploratory Aims

The here-mentioned exploratory aims were not predefined but later on added to further improve and clarify the aforementioned aims.

##### Incidence of GenAI-hallucinations

To explore the incidence of GenAI-hallucinations, we screened question 1 and question 2 of each AI tool. Hallucinations were defined as the generation of non-existent or unverifiable publications; interpretive inaccuracies regarding article content or retraction reasons were not included in this definition. This screening was limited to checking the existence of each link and, when possible, comparing the trial details of each article described to the trial details of the corresponding link. Cases in which we found errors (like incorrect first author, incorrect journal,..) were recorded but not judged as hallucination since the respective article exists.

##### Nonretracted Control Group

We retrospectively added a control group of nonretracted articles. This was deemed necessary to give an estimate on the ability of AI tools to find articles when provided with a list of keywords. Keywords were generated in the same fashion as described in the methods above; we only asked question 1 and question 2.

### Analysis

#### Data Extraction

Data were gathered in a predefined Microsoft Excel file with full-text answers and ratings. This process was performed by two independent researchers (MN and SL). After completion of the data extraction, results were compared between authors and, in case of uncertainty, discussed in the author group. After each article (with the 5 respective questions), a new chat session was opened.

#### Statistical Analysis

Descriptive statistics were used to summarize the performance of each AI tool across all retracted articles and questions. For each tool, the proportion of correct answers was calculated separately for each of the five predefined questions as well as across all questions combined. Results were reported as absolute counts and percentages.

To address the primary outcome, the number of retracted articles for which an AI tool provided correct answers to all five questions was determined. This was expressed as frequency and percentage of the total.

For secondary outcomes, error rates were calculated as follows:

1. Inclusion of retracted articles in topic overviews (Questions 1 and 2): Proportion of instances where a retracted article was mentioned without retraction warning.
2. Missed retraction status in search pathway (Questions 1–3): Proportion of instances in which a retracted article was included without an explicit warning of itsretraction status.
3. Intra-tool consistency: Agreement between duplicate question-answer pairs was assessed by calculating the percentage of conflicting ratings between reviewers for each AI tool.
4. Comparison between general-purpose AI tools and research-focused AI tools: Median number of correct answers per question was compared between the two groups.

Comparisons between general-purpose and research-focused AI tools were treated as exploratory. Differences between groups were summarized using descriptive statistics and effect size estimates, without formal hypothesis testing, due to the limited number of tools per group.

Interreviewer agreement during data extraction was assessed using Cohen kappa coefficient (κ), with percentage agreement reported as a complementary measure.

All analyses were conducted using R (version 4.3.2; R Foundation for Statistical Computing) and Microsoft Excel 2023 for data entry and descriptive summaries.

## Results

### Overview

Data extraction was performed between June and September 2025. A total of 675 prompts were submitted, each asked twice across all models.

**Figure 1.**
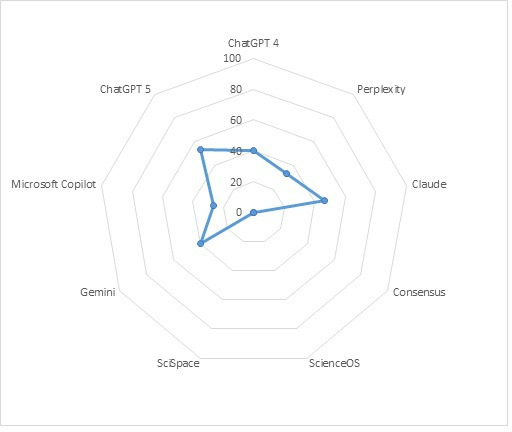
Performance of nine freely available generative artificial intelligence (AI) tools in a pragmatic cross-sectional evaluation of their ability to correctly handle retracted scientific publications. Shown is the proportion of retracted articles (n=15) for which each artificial intelligence tool provided fully correct responses.

The primary aim of this analysis was to assess how often each GenAI tool achieved a perfect score of 5/5 correct answers per question set. None of the evaluated models consistently achieved full accuracy. Three tools - SciSpace, ScienceOS, and Consensus - failed to provide any completely correct responses. The best-performing model was ChatGPT 5, which achieved 8 out of 15 perfect results (53.3%). Model-specific performance data can be seen in Figure 1.

### Retracted Articles in Topic Overviews and Search Pathway

To assess the risk of incorporating retracted studies into literature syntheses, topic overviews and combined topic overviews with article summaries were analyzed separately.

When examining only topic overviews (ie, the first two questions), Microsoft Copilot gave an incorrect answer in 2 (13.3%) out of 15 cases. SciSpace, on the other hand, had the most incorrect cases with 8 out of 15 cases. Exact model performances are presented in the supplementary file figure S1 in Multimedia Appendix 1 and Table 1.

To assess the risk of incorporating retracted studies into literature syntheses, topic overviews, and combined topic overviews with article summaries were analyzed separately.

**Table 1.** Primary analysis of nine freely available generative artificial intelligence tools in a pragmatic cross-sectional study evaluating the handling of retracted scientific literature. For each tool, the table reports the number and percentage of retracted articles (n=15) with fully correct responses to all five predefined questions, the frequency of unflagged inclusion of retracted articles in topic overviews, and intratool reliability based on discrepancies between repeated responses.

GenAI^a^	All 5 correct, n (%)	Retracted article in topic overviews, n (%)	Reliability, n (%)
ChatGPT 4	6 (40)	3 (20)	2 (2.67)
ChatGPT 5	8 (53.34)	3 (20)	5 (6.67)
Microsoft Copilot	4 (26.67)	2 (13.34)	8 (10.67)
Gemini	6 (40)	3 (20)	12 (16)
SciSpace	0 (0)	8 (53.34)	7 (9.33)
ScienceOS	0 (0)	6 (40)	8 (10.67)
Consensus	0 (0)	6 (40)	19 (25.33)
Claude	7 (46.67)	6 (40)	12 (16)
Perplexity	5 (33.34)	6 (40)	17 (22.67)

a GenAI: generative artificial intelligence.

### Reliability of Tools

Each GenAI tool was asked every question twice to assess consistency. Differences between repeated answers resulting in a rating change occurred in each GenAI tool. ChatGPT 4 had the lowest variability with only two inconsistent answers (2.67%). Consensus had the highest incidence with 19 differences, approximately 1 in every 4 questions. Detailed results for each AI are shown in Table 1. We additionally calculated Cohen kappa, which ranged between 0.43 in Perplexity to 0.92 in ChatGPT, with an overall Cohen kappa of 0.73 (table S3 in Multimedia Appendix 1)

### Comparison of General versus Scientific GenAI Tools

General-purpose AI tools achieved a median of 6 fully correct responses out of 15 (range 4‐8), corresponding to an aggregated accuracy of 40%. In contrast, research-focused tools did not achieve any fully correct responses (median 0, range 0‐0). The absolute difference in aggregated proportions between groups was 40 percentage points. Given the limited number of tools per group, this comparison is descriptive and exploratory.

### Comparison of Keywords

Keyword overlap was assessed using a moderately generous matching strategy designed to account for realistic linguistic variation while maintaining methodological rigor. Specifically, orthographic variants (eg, “COVID-19” vs “covid19”), singular and plural forms (eg, “risk factor” vs “risk factors”), hyphenation differences (eg, “meta-analysis” vs “meta analysis”), and established abbreviations versus full terms (eg, “RCT” vs “randomized controlled trial,” “AI” vs “artificial intelligence”) were treated as equivalent. In addition, clearly synonymous or lexically overlapping technical terms referring to the same scientific entity or method (eg, “Alzheimer’s disease” vs “Alzheimer pathology,” or “clinical trials” vs “clinical trial”) were considered matches. In contrast, broader conceptual or hierarchical relationships were explicitly excluded from matching; for example, general versus specific terms (eg, “mental health” vs “depression”), different abstraction levels (eg, “hypertension” vs “blood pressure control”), or loosely related thematic concepts (eg, “public health” vs “disease prevention”) were not treated as equivalent. Pairwise comparisons showed substantial keyword overlap across all model pairs, ranging from 68% to 78%. The greatest overlap was observed between Claude and Perplexity (78%), followed by ChatGPT-4 and Perplexity (74%). ChatGPT-4 and Claude, and Gemini and Claude, each demonstrated 72% overlap, while Gemini and Perplexity showed 70% overlap and ChatGPT-4 and Gemini showed the lowest overlap at 68%. Comparable overlap magnitudes are observed both in comparisons involving the reference model and in comparisons among nonreference models, indicating a high degree of thematic convergence across LLMs. This matching strategy was selected to balance linguistic flexibility with analytical conservatism, avoiding both overly strict string-based matching and overly permissive conceptual aggregation. Nonetheless, we want to raise awareness that ChatGPT may have had a benefit as the keywords used for our search were generated with ChatGPT 4 and no perfect overlap was shown between AI tools.

## Discussion

### Principal Findings and Interpretation

In this cross-sectional evaluation of 9 freely available GenAI tools, none were able to consistently recognize or correctly handle retracted scientific publications. Across all 5 predefined research tasks, no model achieved perfect accuracy, and even the best-performing system, ChatGPT 5, correctly processed less than two-thirds of retracted articles (8 of 15; Figure 1). By contrast, AI tools marketed specifically for scientific use—Consensus, SciSpace, and ScienceOS—performed particularly poorly, each failing to produce a single fully correct set of answers. As shown in Table 1, intra-tool variability was observed across all GenAI systems, with discordance rates between repeated queries ranging from 2.67% in ChatGPT 4% to 25.33% in Consensus, indicating that even the most consistent model failed to produce fully stable responses. These findings collectively indicate that current GenAI systems cannot reliably detect or flag retracted literature, even when explicitly queried about retraction status.

**Figure 2.**
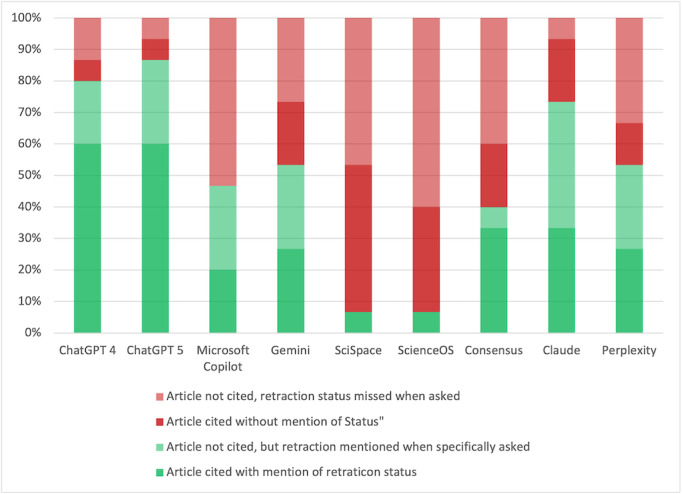
Accuracy of freely available generative artificial intelligence (AI) tools across the first three predefined literature-search tasks: topic overview, identification of relevant articles, and summarization of a specified retracted publication.

The key result of this study is the persistent inability of LLMs to identify retracted papers or to transparently communicate their status to users. This deficiency was observed both in exploratory topic overviews and in direct article queries, suggesting that the problem is systemic rather than situational. As illustrated in Figure 2, the models most frequently cited retracted studies without warning, and even when asked whether a given article had been retracted, many systems returned incorrect statements. These outcomes highlight a fundamental gap in the factual grounding of current AI tools—a limitation that directly undermines their use in scientific research and evidence synthesis. Our analysis cannot determine the exact reason why retracted articles are not marked correctly. Possibilities include the insufficient data retrieval from the original databases (including missing source details or incomplete retrieval) or errors in the reasoning of the respective GenAI. Nevertheless, the scientific end user, who ultimately carries the responsibility for the correctness of his or her article, should be aware of and cautious of this risk.

The contrast between general-purpose and domain-specific AI systems is notable. While one might expect specialized research tools to perform better, they were instead among the weakest performers. Several of these systems rely on static, proprietary databases that are not regularly synchronized with dynamic resources such as PubMed or CrossMark. This architectural rigidity may explain their inability to capture recent retraction events, especially among newly withdrawn papers included in our sample. Conversely, models such as ChatGPT 5 and Claude, which use broader retrieval mechanisms and more frequent model updates, achieved somewhat higher accuracy but still lacked robustness and transparency. Importantly, the results presented represent a performance snapshot of freely accessible AI tools at the time of data collection and may not generalize to subscription-based versions or to future model iterations, which may incorporate improved retrieval mechanisms or retraction-aware features.

### Comparison With Previous Literature

Our findings extend previous work that has documented the use of retracted literature by GenAI. Gu et al [11] reported that ChatGPT produced answers referencing retracted oncology studies, without acknowledging their retraction status, in more than 70% of tested prompts. Similarly, Jan et al [10] and Ge et al [6] have shown that GenAI tools frequently fabricate or misattribute citations during literature summarization tasks. Importantly, these earlier studies typically evaluated individual models or single fields within the scientific literature. The present analysis broadens the evidence base by systematically comparing multiple AI systems across heterogeneous scientific domains, including both general and medical literature. The consistency of failure across all tools underscores that this is not an isolated design flaw but a pervasive, cross-platform limitation in current GenAI architectures.

The continued citation and reuse of retracted work represent a well-recognized threat to research integrity and, in clinical disciplines, may even interfere with patient safety as illustrated by the infamous article of Wakefield et al [13,29,32]. Our results demonstrate that GenAI tools risk amplifying this long-standing problem by automating the propagation of unreliable information. Particularly concerning is the tendency of GenAI tools to reproduce incorrect content with high linguistic confidence, which may give users a false sense of authority and discourage verification. Without built-in mechanisms to cross-check bibliographic metadata or retraction notices, AI-assisted literature synthesis may inadvertently erode rather than enhance scientific reliability.

### Implications for Research and Clinical Practice

Given the accelerating adoption of AI systems in academic and clinical workflows, these findings have immediate practical implications. Researchers increasingly use GenAI tools to generate literature summaries, draft background sections, and identify relevant studies. In medicine and health sciences, where evidence accuracy is critical, the unrecognized inclusion of retracted data could distort systematic reviews, meta-analyses, and ultimately clinical recommendations. As such, reliance on unverified AI output in research and clinical contexts may indirectly contribute to patient harm by propagating retracted or unreliable evidence.

Importantly, expectations regarding the handling of retracted literature may reasonably differ depending on the intended purpose of an AI system. For AI tools explicitly designed to support scientific research or evidence synthesis, excluding retracted publications by default or clearly flagging their status aligns with established principles of research integrity and evidence-based practice. In contrast, for general-purpose AI systems, outright exclusion may not always be appropriate, as retracted articles can still be relevant for historical, methodological, or meta-scientific purposes. In such cases, clearly communicating the retraction status to users may represent a more practical and responsible design approach. Given that retracted information cannot be removed from a trained LLM, handling is more realistically achieved through post-generation checks rather than through changes to the underlying model itself.

To mitigate this risk, several structural measures should be prioritized. AI developers should integrate real-time bibliographic verification pipelines using authoritative sources such as Crossref or PubMed Retraction Notices. AI interfaces intended for academic use should provide transparent confidence indicators or citation provenance, enabling users to identify potentially unreliable results. Journals and peer reviewers should require explicit disclosure of any AI assistance in literature searches and verify cited sources independently. These steps are consistent with recent policy recommendations emphasizing “responsible AI use” and “model interpretability” in scientific contexts [33]. These recommendations align with JMIR editorial policies and ICMJE guidance, which emphasize transparent disclosure of AI use, author responsibility for content accuracy, and independent verification of cited sources.

### Strengths and Limitations

This study offers a pragmatic, reproducible evaluation of AI performance under conditions that mirror real-world research behavior. By using predefined and repeated queries on a diverse set of retracted papers, the design captured both intermodel variability and intramodel inconsistency (Table 1). The inclusion of the 10 most-cited and 5 most-recently retracted papers provided a balanced sample, revealing that both historically influential and newly withdrawn studies were equally mishandled.

Nonetheless, several limitations merit consideration. First, the study was confined to freely accessible versions of each AI tool, excluding subscription models that may have more advanced retrieval capabilities and was taken at a specific point in time. Second, while the test set was broad, it remains a small subset of the retraction landscape and may not generalize to all fields, especially since the majority of articles (13/15, 86.7%) were medical articles. While this reflects the intended application of many AI-assisted literature workflows, the predominance of biomedical publications in our sample may limit the generalizability of these findings to other scientific fields, where differing publication cultures, citation structures, and retraction practices could influence AI performance. Third, AI models evolve rapidly; the specific results reported here represent a snapshot of performance as of mid-2025 and may not reflect future iterations. Fourth, given the limited number of retracted articles (n=15), the study was not powered to detect small-to-moderate differences between tools, and nonsignificant results should therefore be interpreted cautiously due to the risk of type II error. Accordingly, between-tool comparisons were considered exploratory and intended to describe observed performance patterns rather than to support confirmatory claims. Finally, the binary scoring approach (correct vs incorrect) captures factual accuracy but does not account for nuances such as ambiguous phrasing (heavily criticized and scrutinized). While distinguishing whether AI systems correctly identify the specific reasons for retraction could provide additional nuance, our analysis intentionally focused on the more fundamental step of detecting and flagging retracted literature, as failure at this stage already represents a critical risk for research integrity.

These factors should be addressed in future validation studies.

This study has several methodological flaws. One being that our way of questioning needed a uniform, comparable way. This was achieved by combining the keywords in a single prompt string, which could differ from a prompt by a common user searching for a specific topic. Furthermore, there are multiple ways why a retracted article was not cited by an AI. This could be due to the fact that the AI decided against citing it, due to an incomplete or outdated database, due to the fact that this article does not report data of interest, or that there are better articles describing similar aspects. In conclusion, we cannot specifically differentiate between all of those possibilities and therefore decided to create real-world judgment in which we just decide whether this article was included without warning or not. We additionally created an exploratory control group in which we used publication-order matched articles to estimate the ability of each AI to detect a specific article. While the results showed that AI tools found the respective article in up to 90% of cases, we want to highlight the limitation that this extraction was performed in newer iterations of each AI tool.

### Future Directions

As GenAI becomes increasingly embedded in research ecosystems, systematic benchmarking and transparent reporting of factual reliability are essential. Future studies should expand sample sizes, include subscription-based models, and explore automated cross-validation with bibliometric databases. In parallel, developers should implement retraction-aware pipelines capable of dynamic metadata synchronization. Collaborative efforts between publishers, AI developers, and database curators could enable “AI integrity layers” that automatically flag or exclude retracted content before output generation. Ultimately, ensuring that AI systems respect the integrity of the scientific record is both a technical and ethical imperative.

### Conclusions

No currently available free-access GenAI system can be considered reliable for detecting or handling retracted literature. Even the best-performing models failed to consistently recognize the retraction status of scientific articles, while research-focused tools performed particularly poorly. As AI tools become ubiquitous in the preparation and evaluation of scientific work, independent verification of sources and explicit retraction checks remain indispensable to maintain research integrity.

## Supplementary material

10.2196/88766 Multimedia Appendix 1 Supplementary digital content.

## Abbreviations

AI: artificial intelligence
GenAI: generative artificial intelligence
LLM: large language model
