# Supplementary material for: Performance of AI Tools in Citing Retracted Literature : Content Analysis
Source: J Med Internet Res. 2026 May 1;28:e88766. doi: 10.2196/88766 (PMC13134821; doi:10.2196/88766)
Supplement: Multimedia Appendix 1 [file jmir-v28-e88766-s001.docx]

**Supplementary Digital Content -** **Performance of AI tools in citing retracted literature**

[Supplemental table 1: Retracted articles 2](#_Toc225061294)

[Supplementary figure S1: Retracted articles in topic overviews 7](#_Toc225061295)

[Supplementary figure S2: Reliability 8](#_Toc225061296)

[Supplementary table S2 – Retracted article in search pathway 9](#_Toc225061297)

[Supplementary table S3: Cohen’s kappa 10](#_Toc225061298)

[Supplement - AI-hallucinations 11](#_Toc225061299)

[Supplement - Non-retracted control group 12](#_Toc225061300)

[Supplement - OpenEvidence 18](#_Toc225061301)

# Supplementary Table S1: Retracted articles

| **Title** | **Authors** | **Journal, Year of publication** | **Keywords used in the analysis** |
| --- | --- | --- | --- |
| **Most cited, retracted articles** | | |  |
| Pluripotency of mesenchymal stem cells derived from adult **marrow.** | Jiang Y, Jahagirdar BN, Reinhardt RL et al. | Nature, 2002 | Mesenchymal stem cells (MSCs)  Multipotent adult progenitor cells (MAPCs)  Pluripotency  Bone marrow-derived stem cells  In vitro differentiation  Germ layer differentiation  Blastocyst injection  Hematopoietic engraftment  Tissue-specific differentiation  Chimerism |
| Hydroxychloroquine and azithromycin as a treatment of COVID-19: results of an open-label non-randomized clinical trial. | Gautret P, Lagier JC, Parola P, et al. | Int J Antimicrob Agents, 2020 | COVID-19  SARS-CoV-2  Hydroxychloroquine  Azithromycin  Antiviral therapy  Clinical trial  Virological clearance  Drug repurposing  Nasopharyngeal viral load  Non-randomized study |
| 6-month consequences of COVID-19 in patients discharged from hospital: a cohort study. | Huang C, Huang L, Wang Y, et al. | Lancet, 2021 | COVID-19  SARS-CoV-2  2019-nCoV  Clinical features  Wuhan outbreak  Pneumonia  Respiratory symptoms  Intensive care  Viral infection  Coronavirus disease |
| Primary prevention of cardiovascular disease with a Mediterranean diet. | Estruch R, Ros E, Salas-Salvadó J, et al. | N Engl J Med, 2013 | Mediterranean diet  Cardiovascular disease  Primary prevention  Randomized controlled trial  Nutritional intervention  Heart disease prevention  Diet and health  PREDIMED study  Monounsaturated fats  Olive oil and nuts |
| A specific amyloid-beta protein assembly in the brain impairs memory. | Lesné S, Koh MT, Kotilinek L, et al. | Nature, 2006 | Amyloid-β oligomers  Ab*56  Alzheimer’s disease  Memory impairment  Tg2576 mice  Soluble amyloid assemblies  Synaptic dysfunction  Neurodegeneration  Preclinical Alzheimer’s  Morris water maze |
| Predictive validity of a medication adherence measure in an outpatient setting. | Morisky DE, Ang A, Krousel-Wood M, et al. | J Clin Hypertens (Greenwich), 2008 | Medication adherence  Hypertension  Self-reported adherence  8-item Morisky scale  Blood pressure control  Psychometric validation  Outpatient care  Predictive validity  Patient compliance  Health behavior assessment |
| MicroRNA signatures of tumor-derived exosomes as diagnostic biomarkers of ovarian cancer. | Taylor DD, Gercel-Taylor C. | Gynecol Oncol, 2008 | Ovarian cancer  Exosomes  MicroRNA biomarkers  Tumor-derived exosomes  Early cancer detection  EpCAM  Liquid biopsy  Non-invasive diagnostics  Cancer screening  Exosomal microRNA profiling |
| Ileal-lymphoid-nodular hyperplasia, non-specific colitis, and pervasive developmental disorder in children. | Wakefield AJ, Murch SH, Anthony A, et al. | Lancet, 1998 | Autism spectrum disorder  Developmental regression  Gastrointestinal inflammation  Ileal lymphoid hyperplasia  Non-specific colitis  Pervasive developmental disorder  MMR vaccine  Neuropsychiatric symptoms  Pediatric gastroenterology  Urinary methylmalonic acid |
| **Recent progress in processing and properties of ZnO.** | Pearton SJ, Norton DP, Ip K, et al. | Progress in Materials Science, 2005 | Zinc oxide (ZnO)  Semiconductor processing  Wide bandgap materials  Transparent conducting oxides  Thin-film deposition  Doping and defect engineering  Optoelectronic devices  P-type ZnO  Ion implantation  Surface passivation |
| Visfatin: a protein secreted by visceral fat that mimics the effects of insulin. | Fukuhara A, Matsuda M, Nishizawa M, et al. | Science, 2005 | Visfatin  Pre-B cell colony-enhancing factor (PBEF)  Insulin mimetic  Adipocytokine  Visceral fat  Glucose metabolism  Insulin receptor activation  Adipocyte differentiation  Type 2 diabetes  Metabolic syndrome |
| **Newest retracted articles** | | |  |
| Detection of SARS-CoV-2 from patient fecal samples by whole genome sequencing. | Papoutsis A, Borody T, Dolai S, et al. | Gut Pathog, 2021 | **SARS-CoV-2**  **COVID-19**  **Fecal–oral transmission**  **Stool sample diagnostics**  **Next-generation sequencing (NGS)**  **Whole genome sequencing**  **Viral mutation analysis**  **Gastrointestinal shedding**  **RT-PCR vs. NGS**  **Hydroxychloroquine treatment** |
| Surface Hydrophilic Modification for Chip of Centrifugal Microfluidic Immunoassay System. | Shi Y, Ye P, Wang C, et al. | Micromachines, 2022 | **Biosensors**  **Breast cancer**  **Tumor markers**  **Point-of-care diagnostics**  **Electrochemical biosensors**  **Microfluidics**  **Lab-on-a-chip**  **Cancer biomarker detection**  **Immunosensors**  **Nanotechnology in diagnostics** |
| Prevalence and knowledge of polycystic ovary syndrome (PCOS) and health-related practices among women of Syria: a cross-sectional study. | Bohsas H, Alibrahim H, Swed S, et al. | J Psychosom Obstet Gynaecol, 2024 | P**olycystic ovary syndrome (PCOS)**  **Women's health**  **Public health awareness**  **Reproductive health**  **Health education**  **Cross-sectional study**  **Prevalence study**  **Syria**  **Knowledge and practices**  **Gynecological disorders** |
| Burnout components, perceived stress and hair cortisol in healthcare professionals during the second wave of COVID 19 pandemic. | Fortuna F, Gonzalez D, Fritzler A, et al. | Sci Rep, 2024 | **Burnout syndrome**  **Hair cortisol**  **Healthcare workers**  **Chronic stress**  **COVID-19 pandemic**  **Maslach Burnout Inventory (MBI)**  **Perceived stress**  **Social support**  **Biomarkers of stress**  **Depersonalization** |
| A non-enzymatic, isothermal strand displacement and amplification assay for rapid detection of SARS-CoV-2 RNA. | Mohammadniaei M, Zhang M, Ashley J, et al. | Nat Commun, 2021 | **SARS-CoV-2 Lambda variant**  **COVID-19 variants**  **Immune escape**  **Neutralizing antibodies**  **Viral infectivity**  **Spike protein mutations**  **Vaccine resistance**  **Pseudovirus assay**  **ACE2 binding**  **Variant of interest (VOI)** |

Retracted articles used in this investigation

# Supplementary figure S1: Retracted articles in topic overviews

Percentage of incorrect topic overviews (Questions 1 and 2)

# Supplementary figure S2: Reliability

Percentage of questions with different answers (i.e. different ratings between authors) when asked twice

# Supplementary table S2 – Retracted article in search pathway

| **Generative AI** | **Article cited, Retraction status not mentioned (n, %)** | **Article cited, Retraction status mentioned (n, %)** | **Article not cited, retraction status mentioned (n, %)** | **Article not cited, retraction status not mentioned (n, %)** |
| --- | --- | --- | --- | --- |
| **ChatGPT 4** | 1 (6.67) | 9 (60.00) | 3 (20.00) | 2 (13.34) |
| **ChatGPT 5** | 1 (6.67) | 9 (60.00) | 4 (26.67) | 1 (6.67) |
| **Microsoft Copilot** | 0 (0) | 3 (20.00) | 4 (26.67) | 8 (53.34) |
| **Gemini** | 3 (20.00) | 4 (26.67) | 4 (26.67) | 4 (26.67) |
| **SciSpace** | 7 (46.67) | 1 (6.67) | 0 (0) | 7 (46.67) |
| **ScienceOS** | 5 (33.34) | 1 (6.67) | 0 (0) | 9 (60.00) |
| **Consensus** | 3 (20.00) | 5 (33.34) | 1 (6.67) | 6 (40.00) |
| **Claude** | 3 (20.00) | 5 (33.34) | 6 (40.00) | 1 (6.67) |
| **Perplexity** | 2 (13.34) | 4 (26.67) | 4 (26.67) | 5 (33.34) |

Accuracy of freely available generative artificial intelligence (AI) tools across the first three predefined literature-search tasks: topic overview, identification of relevant articles, and summarization of a specified retracted publication. The analysis was based on responses related to 15 retracted articles (ten most cited and five most recently retracted) identified from the Retraction Watch database. All tools were assessed using standardized prompts with repeated questioning between June and September 2025.

# Supplementary table S3: Cohen’s kappa

| **Model** | **Number of items** | **Cohen’s kappa** |
| --- | --- | --- |
| ChatGPT 4 | 75 | 0.92 |
| ChatGPT 5 | 75 | 0.70 |
| Claude | 75 | 0.57 |
| Consensus | 75 | 0.50 |
| Gemini | 75 | 0.50 |
| Microsoft Copilot | 75 | 0.74 |
| Perplexity AI | 75 | 0.43 |
| ScienceOS | 75 | 0.77 |
| Scispace | 75 | 0.80 |

# Supplementary table - AI-hallucinations

Overall, the generative AI tools provided between 6 and 39 links per answer. Details on the number of errors and hallucinations per retracted article are provided in table S4.

Table S4: AI-hallucinations

| **Model** | **Hallucinations** (n, %) | **Errors** (n, %) |
| --- | --- | --- |
| ChatGPT 4 | 7 (46.67) | 3 (20.00) |
| ChatGPT 5 | 7 (46.67) | 4 (26.67) |
| Claude | 4 (26.67) | 0 (0) |
| Consensus | 0 (0) | 6 (40.00) |
| Gemini | 4 (26.67) | 5 (33.34) |
| Microsoft Copilot | 5 (33.34) | 4 (26.67) |
| Perplexity AI | 5 (33.34) | 2 (13.34) |
| ScienceOS | 0 (0) | 3 (20.00) |
| Scispace | 2 (13.34) | 1 (6.67) |

Hallucinations and errors are reported per retracted article analysed, and as relative to total number of articles (n=15)

# Supplement Table - Non-retracted control group

A possible confounder of our results is that answers in which the cited article did not exist were judged equally to questions in which the retraction was specifically mentioned. To gather further insight into this topic, we added a control group consisting of non-retracted articles. Ideally, this group would have consisted of articles with the same context, of similar importance in the field and published on a similar date. Identifying articles, that precisely fit this description is virtually impossible. We therefore decided to use publication-order matched controls. These articles were published directly before or after the retracted article and listed in Table S5.

Table S5: Articles for control group

| **Title** | **Authors** | **Journal, date of publication** | **Generated keywords** |
| --- | --- | --- | --- |
| Dopamine neurons derived from embryonic stem cells function in an animal model of Parkinson's disease. | Kim, JH., Auerbach, J., Rodríguez-Gómez, J. et al. | Nature, 2002 | Parkinson’s disease Dopamine neurons Embryonic stem cells Cell transplantation Neurodegeneration Animal model Neural differentiation Functional recovery |
| Effect of initial antifungal therapy on mortality among patients with bloodstream infections with different Candida species and resistance to antifungal agents: A multicentre observational study by the Turkish Fungal Infections Study Group. | Doğan Ö, Yeşilkaya A, Menekşe Ş, et al. | Int J Antimicrob Agents, 2020 | Candidemia Candida species Antifungal therapy Antifungal resistance Mortality Bloodstream infections Multicentre observational study Clinical outcomes |
| Burden of disease attributable to unsafe drinking water, sanitation, and hygiene in domestic settings: a global analysis for selected adverse health outcomes. | Wolf J, Johnston RB, Ambelu A, et al. | Lancet, 2023 | Unsafe drinking water Sanitation and hygiene (WASH) Global disease burden Environmental health Adverse health outcomes Domestic settings Population health Risk attribution Global health analysis |
| Combination antifungal therapy for cryptococcal meningitis. | Day JN, Chau TTH, Wolbers M, et al. | N Engl J Med, 2013 | Cryptococcal meningitis Combination antifungal therapy Amphotericin B Flucytosine Antifungal treatment outcomes Randomized controlled trial HIV-associated infections Central nervous system fungal infections |
| The finished DNA sequence of human chromosome 12. | Scherer SE, Muzny DM, Buhay CJ, et al. | Nature, 2006 | Human chromosome 12 Genome sequencing Finished DNA sequence Human genome Comparative genomics Gene annotation Chromosomal structure Genomic architecture |
| Impact of terminal digit preference by family physicians and sphygmomanometer calibration errors on blood pressure value: implication for hypertension screening | Niyonsenga T, Vanasse A, Courteau J, et al. | J Clin Hypertens (Greenwich), 2008 | Blood pressure measurement Terminal digit preference Sphygmomanometer calibration Hypertension screening Measurement error Primary care Clinical accuracy Cardiovascular risk assessment |
| Adnexal masses: accuracy of detection and differentiation with multidetector computed tomography | Tsili AC, Tsampoulas C, Charisiadi A, et al. | Gynecol Oncol, 2008 | Adnexal masses Multidetector computed tomography Ovarian tumors Pelvic imaging Diagnostic accuracy Benign and malignant differentiation Gynecologic oncology Cross-sectional imaging |
| Primary symptomless colonisation by Clostridium difficile and decreased risk of subsequent diarrhoea. | Shim JK, Johnson S, Samore MH, et al. | Lancet, 1998 | Clostridium difficile Asymptomatic colonization Antibiotic-associated diarrhea Risk reduction Gastrointestinal microbiology Host–pathogen interaction Intestinal colonization Clinical epidemiology |
| Modelling the semisolid processing of metallic alloys | H.V. Atkinson | Progress in Materials Science, 2004 | Semisolid processing Metallic alloys Thixotropy Rheology Microstructural evolution Solid–liquid coexistence Constitutive modeling Materials processing |
| Mathematical modeling of planar cell polarity to understand domineering nonautonomy. | Amonlirdviman K, Khare NA, Tree DR, et al. | Science, 2005 | Planar cell polarity Domineering nonautonomy Mathematical modeling Computational biology Developmental signaling Tissue patterning Cell–cell interactions Systems biology |
| Understanding the etiology of diarrheal illness in Cambodia in a case-control study from 2020 to 2023. | Kietsiri P, Sornsakrin S, Nou S, et al. | Gut Pathog, 2021 | Diarrheal illness Etiology Case-control study Cambodia Infectious diseases Gastrointestinal pathogens Epidemiology Public health |
| Design and Modeling of a Microfluidic Coral Polyps Culture Chip with Concentration and Temperature Gradients. | Zhou S, Fu ES, Chen B, et al. | Micromachines, 2022 | Microfluidic chip Coral polyps culture Gradient generation Temperature control Concentration gradient Lab-on-a-chip Computational modeling Marine biotechnology |
| Associations between patterns of social support and perinatal mental health among Chinese mother: the mediating role of social trust. | Sun S, An S. | J Psychosom Obstet Gynaecol, 2024 | Perinatal mental health Social support Social trust Chinese mothers Maternal well-being Psychosocial factors Pregnancy Postpartum outcomes |
| Impact of post RFA treatment on neosquamous epithelium microstructure. | Skrobic O, Simic A, Pesko P, et al. | Sci Rep, 2024 | Radiofrequency ablation (RFA) Neosquamous epithelium Microstructure analysis Esophageal treatment Tissue remodeling Histopathology Post-procedural outcomes Gastroenterology |
| Subpicosecond metamagnetic phase transition in FeRh driven by non-equilibrium electron dynamics. | Pressacco F, Sangalli D, Uhlíř V, et al. | Nat Commun, 2021 | FeRh alloy Metamagnetic phase transition Subpicosecond dynamics Non-equilibrium electron dynamics Ultrafast magnetism Spintronics Time-resolved spectroscopy Condensed matter physics |

Table S6: Non-retracted articles identified

| Model | Article identified* (n, %) | Number of correct identifications**(n, %) |
| --- | --- | --- |
| ChatGPT 5 | 14 (93.34) | 24 (80.00) |
| Claude | 11 (73.34) | 19 (63.34) |
| Consensus | 12 (80.00) | 22 (73.34) |
| Gemini | 11 (73.34) | 18 (60.00) |
| Microsoft Copilot | 11 (73.34) | 19 (63.34) |
| Perplexity AI | 11 (73.34) | 21 (70.00) |
| ScienceOS | 12 (80.00) | 20 (66.67) |
| Scispace | 13 (86.67) | 25 (83.34) |

* The article was cited in question 1 or question 2, percentages are calculated relative to the total amount of control group articles (15 articles; OpenEvidence 12 articles)

** relative value is calculated to the total questions

The results of this control group were generated in January 2026. Overall, several limitations of this control group should be considered. Firstly, AI tools undergo continuous development, therefore these results may have differed if extracted in the same timeframe as our main group. Secondly it is not possible to obtain a fully equivalent control group, as the influence of each article on topic coverage varies. This cannot be controlled for and is additionally aggravated that recent research is more likely to be of interest as methods may have change over time. The results presented should therefore be interpreted as a rough estimate rather than accurate and precise.

# Supplement - OpenEvidence

OpenEvidence, a generative AI tool developed for healthcare professionals was also included in our analysis. It is limited to healthcare and therefore articles from other disciplines were out of scope (2 out of 15 articles of our primary dataset), resulting in 65 prompts for analysis of OpenEvidence. We report the results descriptively without comparison to the other generative AI tools.

We found no hallucinations or errors in the answers provided with OpenEvidence.

OpenEvidence was also used for the non-retracted control group. Within this group, three articles were therefore outside the medical scope of OpenEvidence. Detailed results for OpenEvidence are presented in Table S7 and S8:

Table S7 – Results OpenEvidence

|  | **5/5 correct**  **n (%)** | **Retracted article in topic overviews n (%)** | **Search approach retraction missed n (%)** | **Reliability**  **n (%)** | **Cohen’s kappa** |
| --- | --- | --- | --- | --- | --- |
| **OpenEvidence** | 2 (15.38) | 0 (0) | 0 (0) | 3 (4.62) | 0.91 |

*The table reports the number and percentage of retracted articles (n=13) with fully correct responses to all five predefined questions, the frequency of unflagged inclusion of retracted articles in topic overviews, the frequency of missed retraction status, and intra-tool reliability based on discrepancies between repeated responses. Rtracted articles were selected from the Retraction Watch database.*

Table S8 – Non-retracted control-group

|  | Article identified* (n, %) | Number of correct identifications**(n, %) |
| --- | --- | --- |
| OpenEvidence | 7 (58.34) | 14 (58.34) |

* The article was cited in question 1 or question 2, relative to the total amount of control group articles (12 articles, as three articles were out of scope for OpenEvidence)

** relative value is calculated to the total questions
